# Melanization in Cryptococcus neoformans Requires Complex Regulation

**DOI:** 10.1128/mBio.03313-19

**Published:** 2020-02-04

**Authors:** Radames J. B. Cordero, Emma Camacho, Arturo Casadevall

**Affiliations:** a Department of Molecular Microbiology and Immunology, Johns Hopkins Bloomberg School of Public Health, Baltimore, Maryland, USA

**Keywords:** Bzp4, *Cryptococcus neoformans*, Gsk1, MRC-TF, Mbs1, Usv101, laccase, melanin, melanin regulation, melanin-regulating core transcription factors, melanization, nutrient starvation response

## Abstract

The fungal human pathogen Cryptococcus neoformans undergoes melanization in response to nutrient starvation and exposure to exogenous melanin precursors. Melanization protects the fungus against host defense mechanisms such as oxidative damage and other environmental stressors (e.g., heat/cold stress, antimicrobial compounds, ionizing radiation). Conversely, the melanization process generates cytotoxic intermediates, and melanized cells are potentially susceptible to overheating and to certain melanin-binding drugs. Despite the importance of melanin in C. neoformans biology, the signaling mechanisms regulating its synthesis are poorly understood. The recent report by D. Lee, E.-H. Jang, M. Lee, S.-W. Kim, et al. [mBio 10(5):e02267-19, 2019, https://doi.org/10.1128/mBio.02267-19] provides new insights into how C. neoformans regulates melanization. The authors identified a core melanin regulatory network consisting of transcription factors and kinases required for melanization under low-nutrient conditions. The redundant and epistatic connections of this melanin-regulating network demonstrate that C. neoformans melanization is complex and carefully regulated at multiple levels. Such complex regulation reflects the multiple functions of melanin in C. neoformans biology.

## Abstract (precis)

The fungal human pathogen Cryptococcus neoformans undergoes melanization in response to nutrient starvation and exposure to exogenous melanin precursors. Melanization protects the fungus against host defense mechanisms such as oxidative damage and other environmental stressors (e.g., heat/cold stress, antimicrobial compounds, ionizing radiation).

## COMMENTARY

**FIG 1:**
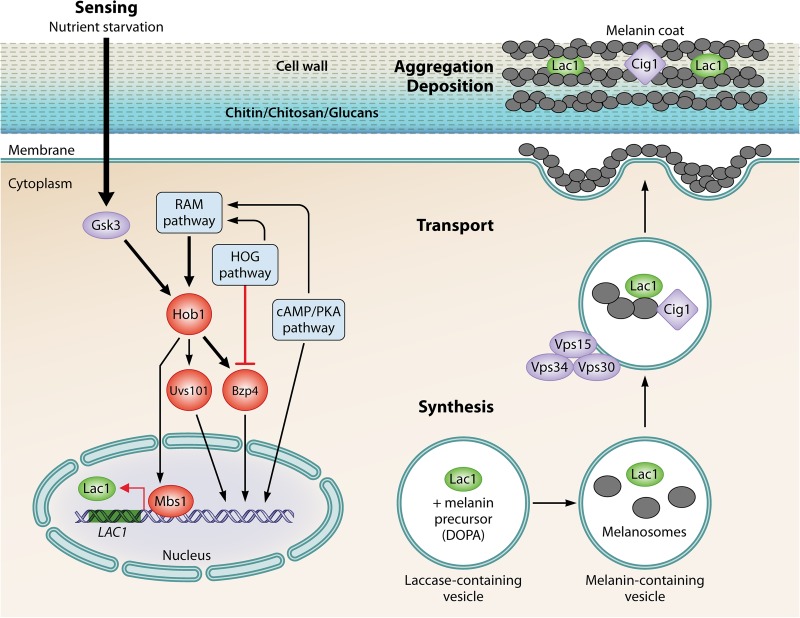
Regulation of C. neoformans melanization. A melanin-regulatory signaling network consisting of four core transcriptional factors (Hob1, Bzp4, Usv101, and Mbs1) is required for C. neoformans melanization via the expression of *LAC1* under low-nutrient conditions and the presence of a melanin precursor (e.g., 3,4-dihydroxyphenylalanine [DOPA]). Each of the MRC-TFs induces *LAC1* expression and modulates epistatic interactions between the cAMP, RAM, and HOG signaling pathways. The Gsk3 kinase induces all four MRC-TFs. The MRC-TFs also govern complex epistatic interactions involved in the multistep process of C. neoformans melanization, from sensing nutritional stress to melanin synthesis, transport, and in the aggregation/deposition of a melanin coat within the cell wall. The Vsp15-Vsp30-Vsp34 complex likely plays important roles in melanin synthesis and transport. The mannoprotein Cig1 physically interacts with melanin granules found in the extracellular environment (1), and its function is associated with iron homeostasis. MRC-TFs also regulate proteins involved in regulating chitin metabolism, which is required for melanin deposition within the cell wall. For a detailed model representing the melanin regulatory signaling network, refer to the original research article by Lee et al. (10).

Melanin serves diverse functions in Cryptococcus neoformans biology, mainly associated with adaption to diverse environmental stress conditions. Like mammalian melanization, the process of melanization in C. neoformans is complex and involves the coordination of multiple steps, including the synthesis, transport, aggregation, and deposition of melanin granules at the inner cell wall (1) (Fig. 1). Melanin synthesis takes place within intracellular vesicles or melanosomes containing a laccase enzyme (2). C. neoformans laccases catalyze melanin polymerization, which involves a series of reduction and oxidation (redox) reactions. These melanin-containing vesicles are then transported across the plasma membrane to the fungal cell wall to form a melanin coat. The melanin coat is formed by a network consisting of melanin granules linked as concentric layers circling the C. neoformans cell body (3). Melanized C. neoformans cells are more resistant to oxidative damage, acidic conditions, heat/cold stress, antimicrobial compounds, and ionizing radiation (reviewed in references 4 and 5).

Melanization also comes with a cost. Although melanization is associated with protection against a broad range of factors, it may also increase vulnerability under certain conditions. The intermediates during melanin synthesis are free radicals that can be toxic to the cell if not compartmentalized and trafficked controllably. Melanin itself is a free radical that, depending on the physical and chemical environment, may also reduce or oxidize other molecules. The presence of a melanin coat at the cell wall implies that some remodeling occurs during the budding process (6), which may influence growth rates. Melanization alters C. neoformans transcription via repression of genes involved in cellular respiration and growth (7). While melanization protects against amphotericin B and caspofungin, it increases affinity to other drugs such as trifluoperazine (8). C. neoformans melanization increases heat capture from radiation, which can be a growth advantage or disadvantage depending on the ambient temperature and the level of irradiation (9). Since the melanization process in C. neoformans appears to be nonreversible, cell responses must proceed on the basis of the potential costs and benefits of pigment synthesis. The paper by Lee et al. provides a glimpse at the multiple signaling pathways that control the cell’s commitment to melanization (10).

Since C. neoformans laccases catalyze the rate-limiting step in melanin synthesis, melanization is mainly regulated by controlling the expression of laccase enzymes, primarily *LAC1*. Laccase expression and melanization are induced in response to low-nutrient conditions (11, 12). Melanization via laccase activity is also affected by temperature (11, 13), multivalent cations (11, 14, 15), quorum sensing (16, 17), and cell cycle elements (18). Prior reports elucidating the C. neoformans signaling pathways involved in melanization demonstrated that laccase expression is regulated by conserved components of the cyclic AMP/protein kinase A (cAMP/PKA) and high-osmolarity glycerol (HOG) response signaling pathways. While the cAMP/PKA pathway is associated with laccase localization and induction under starvation conditions (19–21), the HOG pathway represses laccase (22, 23). Despite these discoveries, the transcription factors controlling laccase expression were poorly understood.

In their study, Lee et al. performed systematic analyses that combined gene expression and epistatic data from C. neoformans mutant libraries and identified a core consisting of four transcription factors (Bzp4, Hob1, Usv101, and Mbs1) and two kinases (Gsk3 and Kic1) regulating melanin synthesis and other steps during the melanization process (10). Each of the melanin-regulating core transcription factors (MRC-TFs) regulates the expression of the *LAC1* in some distinctive way and mediates interconnections with the cAMP, regulation of Ace2 and morphogenesis (RAM), and HOG signaling pathways (Fig. 1). Whereas Bzp4 and Usv101 demonstrated a defined range of action in terms of the number of genes that they regulate, Hob1 and Mbs1 had pleiotropic roles that affect hundreds of genes. The authors also showed how the MRC-TFs regulate genes involved in vacuole/vesicle trafficking, chitin metabolism, and iron homeostasis. This provides additional levels of regulation, since melanosomes and melanin granules need to be transported and deposited within the cell wall and their deposition involves physical interactions between melanin and chitin (24, 25). Interestingly, the authors showed that Hob1 negatively regulates Cig1, a mannoprotein that is involved in iron homeostasis which was recently found to be physically associated with C. neoformans extracellular melanin granules (1). Together, these findings provide an unprecedented view of how C. neoformans regulates the melanization process at the transcriptional, translational, and posttranslational levels.

From synthesis of melanin to its assembly at the cell wall, the process of C. neoformans melanization requires the coordination of multiple steps and molecular players inside the cell that remain predominantly unknown. Since melanin is involved in different biological functions, it follows that it is regulated by multiple different signaling pathways. The context-specific physiological advantages and disadvantages associated with C. neoformans melanization support the idea that this process requires complex regulation.

The report presented by Lee et al. opens new opportunities to study eukaryotic stress responses and melanization. The discoveries laid out there will facilitate further studies into how these signaling networks regulate melanin synthesis in response to individual stress factors and how similar melanin signaling networks operate in higher eukaryotes. Understanding the signaling pathways that regulate melanization in C. neoformans and other melanotic fungal pathogens (and where these pathways deviate from those of higher metazoans) will lead to the identification of attractive targets for the development of antifungals.
